# Different skeletal effects of the peroxisome proliferator activated receptor (PPAR)α agonist fenofibrate and the PPARγ agonist pioglitazone

**DOI:** 10.1186/1472-6823-9-10

**Published:** 2009-03-30

**Authors:** Unni Syversen, Astrid K Stunes, Björn I Gustafsson, Karl J Obrant, Lars Nordsletten, Rolf Berge, Liv Thommesen, Janne E Reseland

**Affiliations:** 1 Department of Cancer Research and Molecular Medicine, Norwegian University of Science and Technology, Trondheim, Norway; 2 Department of Endocrinology, St Olav's University Hospital HF, Trondheim, Norway; 3 Department of Gastroenterology, St Olav's University Hospital HF, Trondheim, Norway; 4 Department of Surgery, Yale University School of Medicine, New Haven, USA; 5 Department of Orthopaedics, Malmø University Hospital, Malmø, Sweden; 6 Department of Orthopaedics, Ullevål University Hospital, Oslo, Norway; 7 Section of Medical Biochemistry, Institute of Medicine, University of Bergen, Norway; 8 Sør-Trøndelag University College, Faculty of Food Science and Medical Technology, Trondheim, Norway; 9 Department of Biomaterials, Institute for Clinical Dentistry, University of Oslo, Blindern, Oslo, Norway

## Abstract

**Background:**

All the peroxisome proliferator activated receptors (PPARs) are found to be expressed in bone cells. The PPARγ agonist rosiglitazone has been shown to decrease bone mass in mice and thiazolidinediones (TZDs) have recently been found to increase bone loss and fracture risk in humans treated for type 2 diabetes mellitus. The aim of the study was to examine the effect of the PPARα agonist fenofibrate (FENO) and the PPARγ agonist pioglitazone (PIO) on bone in intact female rats.

**Methods:**

Rats were given methylcellulose (vehicle), fenofibrate or pioglitazone (35 mg/kg body weight/day) by gavage for 4 months. BMC, BMD, and body composition were measured by DXA. Histomorphometry and biomechanical testing of excised femurs were performed. Effects of the compounds on bone cells were studied.

**Results:**

The FENO group had higher femoral BMD and smaller medullary area at the distal femur; while trabecular bone volume was similar to controls. Whole body BMD, BMC, and trabecular bone volume were lower, while medullary area was increased in PIO rats compared to controls. Ultimate bending moment and energy absorption of the femoral shafts were reduced in the PIO group, while similar to controls in the FENO group. Plasma osteocalcin was higher in the FENO group than in the other groups. FENO stimulated proliferation and differentiation of, and OPG release from, the preosteoblast cell line MC3T3-E1.

**Conclusion:**

We show opposite skeletal effects of PPARα and γ agonists in intact female rats. FENO resulted in significantly higher femoral BMD and lower medullary area, while PIO induced bone loss and impairment of the mechanical strength. This represents a novel effect of PPARα activation.

## Background

Peroxisome proliferator activated receptors (PPARs) are nuclear transcription factors that modulate the expression of a variety of genes involved in lipid metabolism and fat storage [1-3]. The PPAR family consists of three subtypes, PPARα, β/δ and γ (with splice variants γ 1, 2 and 3) ubiquitously expressed, but with a tissue specific distribution [1,2]. All PPAR subtypes have been identified in osteoblasts [4] and osteoclasts [5-7]. PPAR ligands include fatty acids, eicosanoids, nonsteroidal inflammatory agents and a heterogeneous class of chemicals [1-3,8]. Thiazolidinediones (TZDs) are PPARγ agonists, currently used for the treatment of insulin resistance and type 2 diabetes mellitus [8], while fibrates are mainly PPARα agonists that are efficient in lowering elevated triglyceride concentrations [9]. TZDs have been shown to inhibit osteoclast differentiation and bone resorption [7,10,11], while others report a stimulatory effect on osteoclastogenesis and bone resorption [12]. The fibrates fenofibrate and bezafibrate have been found to inhibit osteoclastogenesis [11,13]. Previous studies have shown that the PPARγ agonist rosiglitazone decreases bone mass in mice [14,15], and enhances bone loss induced by estrogen deprivation in rats [16]. Moreover, TZDs have been associated with bone loss at the whole body, lumbar spine and trochanter in elderly women, but not men, with type 2 diabetes [17]. Recent studies have revealed that rosiglitazone treatment causes elevated fracture rates in women with type 2 diabetes [18], and also decreases bone formation and bone mineral density (BMD) in healthy postmenopausal women [19].

We have previously demonstrated the presence of leptin and its receptors in human osteoblasts and shown that leptin stimulates osteoblast proliferation, differentiation and mineralization [20,21]. TZD administration to rats has shown to reduce the plasma levels of leptin in spite of increase in fat mass [22,23], which may imply that leptin is involved in the skeletal effects observed after treatment with rosiglitazone [14-16]. In contrast, the plasma levels of the adipokine adiponectin are elevated in rats [24,25], as well as humans receiving TZDs [26]. Adiponectin concentrations have also been reported to be elevated after fenofibrate therapy in patients with hypertriglyceridemia [27].

In the present study we examined the long-term effects of the PPARα agonist fenofibrate and the PPARγ agonist pioglitazone on the rat skeleton *in vivo*, using bone mass measurements, histomorphometry and biomechanical testing. The levels of osteocalcin and fragments of collagen type I in plasma, and leptin and adiponectin levels in plasma and femurs were analyzed. Moreover, the *in vitro *effects of fenofibrate and pioglitazone on cytokine release, proliferation and differentiation of a preosteoblast cell line, and differentiation and activity of human osteoclasts were assessed.

## Methods

Fenofibrate was provided by one of the co-authors, and pioglitazone was kindly provided by Eli Lilly, Norway. Methylcellulose (M7140, Sigma-Aldrich, St.Louis, MO) was used as vehicle for both drugs. Female Fischer-344 rats were purchased from Møllegaard's Breeding Center (Skensved, Denmark).

### Study design

The Animal Welfare Committee at Trondheim University Hospital approved this study. Thirty-five two-month-old female Fischer rats (203 ± 11.5 g) were housed solely in wire-top cages with aspen woodchip bedding from B&K Universal Ltd. Room temperature was 24 ± 1.0°C with a relative humidity of 40%–50% and a 12-h light/dark cycle. The Rat and Mouse Diet of B&K, and tap water were provided *ad libitum*. One group received methylcellulose (control, n = 12), and the other two groups methylcellulose with either fenofibrate (n = 11) or pioglitazone (n = 12), 35 mg/kg body weight daily given for 4 months by gavage. The dose of pioglitazone was chosen on the background of previous studies on the long-term skeletal effects of TZDs in rodents [14,16,28]. The dose of fenofibrate was chosen according to other studies with long-term fenofibrate treatment in rats [29,30].

The rats were weighed at the start and weekly throughout the study. Before all procedures and sacrifice, the animals were anesthetized with 2.0 ml/kg body weight of a combination of fluanison (2.5 mg/ml), fentanyl (0.05 mg/ml), and midazolam (1.25 mg/ml). Blood was collected by heart puncture during the finale anesthezia, and stored at -80°C until assaying. The liver weights were also registered. Both femurs were collected and stored at -80°C for further analyses.

### Dual X-ray absorptiometry (DXA) measurements

Bone mineral density (BMD) and bone mineral content (BMC) in femur and whole body, fat mass and lean mass were measured by DXA in intact animals, using a Hologic QDR 4500A, and small animal software. Measurements were performed in duplicate at the start and the end of the study in intact animals. Coefficients of variation were 0.6% for whole body BMD, 0.7% for femur BMD, 0.5% for whole body BMC, 3.0% for femur BMC, 2.2% for total fat content, and 0.3% for lean body mass.

### Histomorphometry

Histomorphometry of the left femur was performed as described previously and was evaluated by two persons (27). Transverse sections were cut close to the patellar ridge and also 5.0 mm proximal from it. They were grounded to a thickness of 50 um. The sections close to the patellar ridge were used for calculation of trabecular bone volume (TBV%), and proximal sections were used for calculation of cortical thickness. The sections for calculation of TBV% were stained with Goldener. For calculation of TBV% a Merz grid at a magnification of 10 × 10 was used. As many squares as possible, usually about 9–11, were calculated. For calculation of cortical thickness the central point of the medullary canal was identified. By means of an eyepiece the diameters were calculated in four directions with a 45 degrees angle between each. Medullary, cortical and total cross-sectional areas were calculated from the mean values. All histomorphometric measurements were performed blindly.

### Biomechanical testing

The right femurs were thawed in Ringers^® ^solution before mechanical testing of the femoral shaft and the femoral neck was performed. The diaphyses were fractured 18.7 mm from the femoral condyles in three point cantilever bending as previously described [31]. The proximal femur was fixed in a clamp, the cam of the rotating wheel engaged the femoral condyles and a fulcrum positioned anteriorly 18.7 mm from the condyles was the third point of force application. All tests were done at a loading rate of 0.095 radians/second (5.43°/second). The load in the test apparatus, an MTS 858 Mini Bionix^® ^Axial/Torsional Test System (MTS Systems Corporation, Minnesota, USA), was measured with a MTS Test Star TM Sensor Cartridge Force 250 N load cell and registered in MTS Test Star II software. Ultimate moment, ultimate energy absorption, stiffness and deflection were read directly or calculated from the computer recordings [32].

### Assessment of protein in femur

After biomechanical analyses, the right femurs were homogenized by crushing in liquid nitrogen, and 200 mg tissue of each sample was lysed in Trizol (Invitrogen, CA, USA) followed by protein isolation according to the manufacturer's instructions.

### Leptin, adiponectin, insulin, osteocalcin, osteoprotegerin (OPG) and bone resorption marker analyses in rat plasma and protein fraction of rat femur and OPG, RANKL and total protein analyses in medium from MC3T3-L1 cells

Using radioimmunoassay (RIA) (Linco Research, St. Charles, Missouri 63304, USA), leptin and adiponectin levels were quantified in plasma and in the isolated femoral protein fraction, and insulin and OPG levels were measured in plasma. Detection limits were 3.0 pg/ml for leptin, 0.78 ng/ml for adiponectin, 18.6 pg/ml for insulin, and 2.3 pg/ml for OPG. Intra- and interassay variations for all RIAs were < 4.5% and < 9.0%, respectively.

Osteocalcin in plasma was determined by a Rat-MID Osteocalcin enzyme-linked immunosorbent assay kit (Nordic Bioscience Diagnostics A/S, Denmark), according to the manufacturer's protocol. The detection limit was 50 ng/ml, and intra- and interassay variations were 5.0% and 5.5%, respectively. Bone resorption markers in plasma (fragments of type 1 collagen) were analyzed by a RatLaps ELISA kit (Nordic Bioscience Diagnostics A/S) according to the instructions from the manufacturer. The detection limit was 3.0 ng/ml, and intra- and interassay variations were 5.6% and 10.5%, respectively.

The concentration of OPG in the mouse cell line MC3T3-E1 culture media was determined by ELISA as described by the manufacturer (RnD Systems, USA) using anti-mouse OPG-antibody and biotinylated anti-mouse OPG. Detection was performed by labeling with streptavidin-horseradish peroxidase (R&D Systems) and adding 1, 2 phenylenediamine dihydrochloride (OPD) (Dako, Glostrup, Denmark) as substrate. The enzymatic reaction was stopped after 20 min by adding 100 μl of 0.5 M H_2_SO_4 _to each well. The optical density (OD) was measured as absorbance at 490 nm. Recombinant murine OPG was used as standard. The detection limit was 10 pg/ml, and intra-assay and inter-assay variabilities were less than 15% and 9.0%, respectively. The amount of OPG was related to the amount of total protein in each sample. RANKL concentration in culture media was determined by an immunoassay kit for quantitative determination of free sRANKL from mouse and rat (Biomedica, Vienna, Austria) according to the manufacturer's protocol. The amount of RANKL was related to the amount of total protein in each sample. The amount of total protein in media was determined using Sigma Microprotein PR assay kit with Protein Standard Solution Calibrator (Sigma Diagnostics, Dorset, UK) according to the manufacturer's protocol. Analyses were performed using the Cobas Mira chemistry analyzer (Roche Diagnostics, Germany). Intra-assay and inter-assay variabilities were less than 2.4% and 3.2% respectively. Detection range for the assay was 10-2.0 × 10^3 ^mg/l.

### Cells and reagents

MC3T3-E1 (mouse preosteoblasts, ATCC) cells were maintained in α-MEM (Invitrogen) supplemented with 10% fetal calf serum (FCS) (EuroClone, Great Britain), 1 mM Na- pyruvate (Gibco BRL, Life Technologies Ltd, Scotland), 0.1 mg/ml L-glutamine (Gibco) and 10 U/ml penicillin/streptomycin (Gibco).

Commercially available human primary precursor osteoclasts, differentiation medium and an Osteolysis Assay kit (Lonza Walkersville, Inc., MD, USA) were used for *in vitro *assays of human osteoclast differentiation and activity according to the manufacturer's protocol.

### Osteoblast differentiation

To study whether fenofibrate and pioglitazone affected osteoblast differentiation, MC3T3-E1 cells were seeded in 6-wells plates (3.0 × 10^5 ^cells/well) and cultured until confluence (3 days). The cells were then cultured for up to 12 days in α-MEM/10% FCS, with or without fenofibrate or pioglitazone. Medium was changed every second day. The relative mRNA expression of the osteoblast differentiation markers including alkaline phosphatase (ALP), bone sialoprotein (BSP), CD44, collagen 1, osteocalcin and osteopontin was studied. The relative mRNA expression of PPARα, PPARγ and of the adipocyte differentiation gene lipoprotein lipase (LPL) was also determined.

### Proliferation assay

Proliferation was studied using a kit from Roche Molecular Biochemicals, Mannheim, Germany. Briefly, MC3T3-E1 cells (4 × 10^3 ^cells/well) were seeded in 96 well plates, and cultured for 24 h. Cells were washed once with 180 μl serum-free medium, before addition of new medium with or without test substances. After 4 h, 5-bromo-2'-deoxyuridine (BrdU)-labeling solution was added, and the cells were cultured for additional 18 h before incorporation of BrdU was measured as described by the manufacturer. After removing the labeling medium, cells were fixed and genomic DNA denaturized by adding 150 μl FixDenat per well for 30 min at room temperature. FixDenat-solution was removed and 100 μl of peroxidase-conjugated anti-BrdU antibody solution was added per well, followed by incubation at room temperature for 90 min. The cells were washed three times with 200 μl washing solution before 100 μl of substrate Luminol/4-idophenol was added. After 3 min, chemiluminescence was measured (RLU = relative luminescence units) in a micro plate luminometer (Fluoroscan Ascent FL, Labsystems).

### mRNA isolation and cDNA synthesis

Cells were homogenized and lysed in lysis/binding buffer and mRNA was isolated using magnetic beads (oligo (dT)_25_) as described by the manufacturer (Dynal AS, Oslo, Norway). Beads containing mRNA were resuspended in 10 mM Tris-HCl, pH 8.0, and stored at -80°C until use. mRNA-containing solution was applied directly to obtain a first-strand complementary DNA (cDNA) using the iScript cDNA Synthesis Kit with oligo(dT) (Bio-Rad, CA, USA). cDNA samples were diluted 1:2 with nuclease free water.

### Real time PCR quantification

Reactions were performed and monitored using Stratagene's Mx3000P Real-time PCR system. The 2× iQ SYBR Green Supermix was based on iTaq DNA polymerase (Bio-Rad, Oslo, Norway). cDNA samples were analyzed both for the genes of interest and reference genes (β-actin) The amplification program consisted of a preincubation step for denaturation of the template cDNA (5 min, 95°C), followed by 40 cycles consisting of a denaturation step (30 s, 95°C), an annealing step (30 s, 60°C) and an extension step (30 s, 72°C). The Ct value, defined as the number of cycles required to produce a detectable product above background fluorescence, was measured for each sample, and arbitrary units were calculated using standard curves that consisted of serial dilutions of cDNA from a pool of samples or controls containing the highest amounts of the specific gene analyzed. Contamination by genomic DNA was ruled out by performing PCR analysis where RT-enzyme had been omitted in the RT reactions. β-actin RT-PCR was run in duplicates or triplets as control to monitor RNA integrity and to be used for normalization. Specificity of each primer pair was confirmed by melting curve analysis and agarose gel electrophoresis, and PCR products were sequenced for product confirmation. Table 1 show the primer sequences, the expected sizes of the PCR products and gene bank accession numbers used for design of the primer pairs. Intron-spanning primers were designed using the computer software Clone. Data is calculated from standard curves, related to housekeeping gene and presented normalized to untreated controls at each individual time point.

**Table 1:** Primers used in real-time PCR quantification

Gene	Primer Sequence	Specie	Size (bp)	Gene Bank Accession Number
Alcalic phosphatase (ALP)	S 5'-AACCCAGACACAAGCATTCC-3'	Mouse	151	NM_007431.1
	AS 5'-GAGAGCGAAGGGTCAGTCAG-3'			
β-actin	S 5'-CTGGCTCCTAGCACCATGA-3'	Mouse	73	NM_031144.2
	AS 5'-AGGCACCAATCCACACAGA-3'			
Bone Sialoprotein	S 5'-GAAAATGGAGACGGCGATAG-3'	Mouse	141	NM_008318.1
	AS 5'-ACCCGAGAGTGTGGAAAGTG-3'			
CD44	S 5'-CTTCCATCTTGACCCGTTGT-3'	Mouse	175	NM_009851.2
	AS 5'-ACAGTGCTCCTGTCCCTGAT-3'			
Collagen 1	S 5'-AGAGCATGACCGATGGATTC-3'	Mouse	177	NM_007742.3
	AS 5'-CCTTCTTGAGGTTGCCAGTC-3'			
Lipoprotein lipase (LPL)	S 5'-GGATAAGCGACTCCTACTTC-3'	Mouse	198	NM_008509.1
	AS 5'-AGCCAGACTTCTTCAGAGAC-3'			
Osteocalcin	S 5'-CCGGGAGCAGTGTGAGCTTA-3'	Mouse	81	L24431.1
	AS 5'-TAGATGCGTTTGTAGGCGGTC-3'			
Osteopontin	S 5'-GACCACATGGACGACGATG-3'	Mouse	498	BC002113.1
	AS 5'-TGGAACTTGCTTGACTATCGA-3'			
PPARα	S 5'-TGAACAAAGACGGGATG-3'	Mouse	106	NM_011144.2
	AS 5'-TCAAACTTGGGTTCCATGAT-3'			
PPARγ	S 5'-CAGGCTTCCACTATGGAGTT-3'	Mouse	105	NM_011146.1
	AS 5'-TCCGGCAGTTAAGATCACAC-3'			

PPAR = peroxisome proliferator activated receptor

### Osteoclast differentiation and activity

Primary human osteoclast precursor cells and differentiation media containing macrophage colony stimulating factor (M-CSF) and soluble RANKL were obtained from Lonza, and cultured according to the provided protocol. Ten thousand cells per well were seeded in a 96-well plate in differentiation medium. Fenofibrate or pioglitazone (10 nM, 0.1, 1.0 and 10 μM) were added in six parallel wells, and cultured for 12 days before cultures were stained for tartrate-resistant acid phosphatase (TRAP), activity using Naphtol AS-BI phosphate and Fast Garnet in the presence of sodium tartrate, as described by the manufacturer (Sigma-Aldrich, Norway). The number of TRAP positive, multinuclear (3 or more nuclei) cells was counted.

Human osteoclast activity was examined using the Osteolyse Assay kit (Lonza,). An osteolyse plate was seeded with 1.0 × 10^4 ^human osteoclast precursor cells (Lonza) per well in medium containing M-CSF and soluble RANKL and cultured at 37°C for 7 days. Control cells were seeded with or without RANKL and M-CSF. Culture media were changed on day 7, and then cultured for another 1 to 3 days with or without fenofibrate (0.1, 1.0 and 10 μM) or pioglitazone (0.1, 1.0 and 10 μM), with six parallels for each condition. Cell medium supernatants were collected on day 8, 9 and 10, and the release of collagen type I was measured by EIA according to the manufacturer's protocol, with the exception that fluorescence was measured by excitation at 355 nm and emission of 650 nm, instead of 340/615 nm as recommended.

### Statistical analyses

Measurements were performed in duplicates or triplets. Data is expressed as means ± SD. All data were tested for normality with Shapiro-Wilk and D'Agostino & Pearson omnibus normality tests. Normally distributed parameters were tested with two-tailed unpaired Student t-test, or one-way ANOVA with Bonferroni's post test, while parameters that were not normally distributed were tested with Mann-Whitney's two tailed test, or Kruskal Wallis test with Dunn's post test. Significance was assumed at *P*-values lower than 0.05. Correlations between normally distributed data sets were analyzed by Pearson's product-moment correlation coefficient test.

## Results

### Anthropometric data, body composition and liver weights

There were no differences in body weight, fat mass or lean mass at the beginning of the study (data not shown). After 4 months of drug administration, the body weight did not differ between the groups, and no difference in femoral length was observed, indicating absence of effects on longitudinal growth (Table 2). The rats given pioglitazone had significantly higher fat mass (*P *= 0.01 vs. controls, and *P *= 0.0001 vs. the group given fenofibrate). Lean mass was significantly lower in the pioglitazone group compared to controls (*P *= 0.005) and the fenofibrate group (*P *= 0.0001) (Table 2). In the fenofibrate group, fat mass did not differ from controls, while lean mass was significantly higher than in control rats (*P *= 0.006) (Table 2). The mean liver weight was significantly higher in the fenofibrate group compared to controls (9.64 ± 0.85 g and 6.98 ± 0.47 g, respectively, *P *= 0.0001). In the pioglitazone group, the liver weight was significantly lower (5.79 ± 0.39 g), both compared to controls (*P *= 0.0168) and to rats receiving fenofibrate (*P *= 0.0001). Since lean mass also include liver weight, the liver weight was subtracted to correct for this. After this correction, lean mass was still significantly higher in the fenofibrate group (185.3 ± 8.5 g), both compared to the control (176.4 ± 7.6 g, *P *= 0.016) and pioglitazone group (165.6 ± 8.6 g, *P *= 0.0001). Corrected lean mass was significantly lower in the pioglitazone group than in the controls (*P *= 0.008). Four animals died in the pioglitazone group, while none died in the other groups. The cause of death in the pioglitazone group was aspiration in connection with gastric gavage in 3 of the rats, in one of the rats no reason could be found. No differences in the well-being of the animals were, however, observed during the study.

**Table 2:** Body composition, femur length, femur and whole body BMC and BMD

Group	Control (n = 12)	Fenofibrate (n = 11)	Pioglitazone (n = 8)
Body weight (g)	237 ± 14.9	243 ± 9.60	240 ± 8.70
Fat mass (g)	46.8 ± 13.7	41.2 ± 6.94	62.5 ± 8.42^**/###^
Lean mass (g)	184 ± 7.73	195 ± 8.98**	171 ± 8.87^**/###^
Femur length (cm)	33.9 ± 0.44	34.1 ± 0.53	33.5 ± 0.57
Femoral BMC (g)	3.56 ± 0.30	3.78 ± 0.28	3.39 ± 0.26^##^
Femoral BMD (g/cm^2^)	0.245 ± 0.009	0.259 ± 0.001**	0.235 ± 0.001^###^
Whole body BMC (g)	7.23 ± 0.36	7.36 ± 0.26	6.87 ± 0.40^*/##^
Whole body BMD (g/cm^2^)	0.159 ± 0.008	0.157 ± 0.005	0.143 ± 0.002^***/###^

BMC = bone mineral content, BMD = bone mineral density. Data is presented as mean ± SD, *P < 0.05, **P < 0.01, ***P < 0.001 significantly different compared to control, ^##^P < 0.01, ^###^P < 0.001 significantly different compared to fenofibrate-treated group.

### Bone mineral content (BMC) and bone mineral density (BMD)

There were no differences in femoral and whole body BMC or BMD at the beginning of the study (data not shown). After four months of treatment, femur BMC tended to be higher, albeit not significantly, in the fenofibrate group compared to controls, (+ 6.2%, *P *= 0.08), while the pioglitazone group had significantly lower femur BMC compared to the fenofibrate group (-10%, *P *= 0.0067), but not compared to controls (Table 2). Femoral BMD was significantly higher in fenofibrate rats (+ 5.8%, *P *= 0.004), while femoral BMD was similar in controls and pioglitazone treated rats (Table 2). The pioglitazone group had significantly lower femoral BMD than the fenofibrate group (-10%, *P *= 0.0003). Whole body BMC was significantly lower in the pioglitazone group after four months of treatment, relative to controls (-5.2%, *P *= 0.047), and relative to the fenofibrate group (-6.6%, *P *= 0.004). Whole body BMD was significantly lower in the pioglitazone group compared to controls (-9.8%, *P *= 0.0001), and also compared to the fenofibrate group (-8.4%, *P *= 0.0001), while there was no difference between the control group and the fenofibrate group (Table 2).

### Histomorphometry and correlation analyses

Trabecular bone volume in the distal femur was lower in the pioglitazone rats compared to control rats and fenofibrate rats (-27%, *P *= 0.006 and – 25%, *P *= 0.019, respectively), while no difference was found between the fenofibrate and the control group (Table 3). The medullary area in distal femur was significantly higher in the pioglitazone rats compared to the fenofibrate group, (+ 14.8%, *P *= 0.0001), and also significantly higher compared to controls (+ 8.0%, *P *= 0.05), (Table 3). In contrast medullary area was lower, in the fenofibrate group compared to controls (-7.2%, *P *= 0.05). There were no differences in cortical and total areas between the groups (Table 3). For the total material, there was a significant positive correlation between lean mass (corrected for liver weight), femoral BMD (Pearsons r = 0.513, *P *= 0.0001) and cortical area (Pearsons r = 0.673, *P *= 0.008).

**Table 3:** Histomorphometric data for femur

Group	Control (n = 12)	Fenofibrate (n = 11)	Pioglitazone (n = 8)
Trabecular bone Volume (%)	22.2 ± 5.95	21.70 ± 5.72	16.3 ± 1.38^**/#^
Total area (mm^2^)	6.34 ± 0.40	6.35 ± 0.23	6.61 ± 0.30
Cortical area (mm^2^)	3.99 ± 0.29	4.18 ± 0.16	4.05 ± 0.21
Medullary area (mm^2^)	2.35 ± 0.25	2.18 ± 0.11*	2.56 ± 0.16^###^

Data is presented as mean ± SD. *P<0.05, **P < 0.01 significantly different compared to control. ^#^P < 0.05, ^###^P < 0.001 significantly different compared to fenofibrate-treated group.

### Biomechanical testing

The ultimate bending moment, deflection and energy absorption of femoral shafts were significantly lower in the pioglitazone rats compared to controls (*P *= 0.007, *P *= 0.039 and *P *= 0.014), and compared to the fenofibrate group (*P *= 0.019, *P *= 0.0025 and *P *= 0.014), while no difference was found between the fenofibrate group and the controls (Table 4). Biomechanical parameters in the femoral neck did not differ significantly between the groups (Table 4).

**Table 4:** Mechanical properties of femoral neck and shaft

Group	Control (n = 12)	Fenofibrate (n = 11)	Pioglitazone (n = 8)
Moment (Nm × 10^-2^)			
Femoral Neck	50.8 ± 5.17	53.9 ± 5.74	49.2 ± 3.65
Femoral Shaft	48.1 ± 3.33	47.9 ± 3.99	43.0 ± 4.18^**/#^
			
Stiffness (Nm/° × 10^-3^)			
Femoral Neck	0.78 ± 0.10	0.78 ± 0.12	0.73 ± 0.10
Femoral Shaft	1.05 ± 0.18	1.04 ± 0.09	1.04 ± 0.15
			
Deflection (°)			
Femoral Neck	23.1 ± 3.00	24.3 ± 2.67	24.0 ± 3.37
Femoral Shaft	16.6 ± 2.53	16.7 ± 1.37	14.4 ± 1.43^*/##^
			
Energy absorption (J × 10^-2^)			
Femoral Neck	1.19 ± 0.12	1.26 ± 0.14	1.15 ± 0.09
Femoral Shaft	1.12 ± 0.08	1.13 ± 0.09	1.01 ± 0.10^*/#^

Data is presented as mean ± SD. **P < 0.01 significantly different compared to control, ^#^P < 0.05, ^##^P < 0.01 significantly different compared to fenofibrate group.

### Leptin and adiponectin measurements

**Figure 1:**
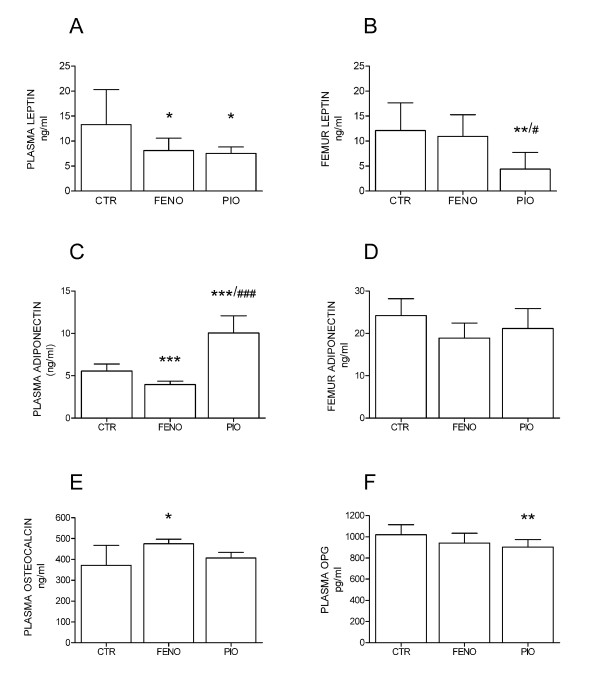
**Plasma (A) and femur (B) levels of leptin (ng/ml), plasma (C) and femur (D) levels of adiponectin (ng/ml), plasma levels of osteocalcin (ng/ml) (E), and plasma levels of osteoprotegerin (OPG) (pg/ml) (F) in control (CTR) rats (n = 12), fenofibrate (FENO) (n = 11) and pioglitazone (PIO)-fed (n = 8) rats after 4 months of daily treatment**. Data is presented as mean ± SD. *P < 0.05, **P < 0.01, ***P < 0.0001 significantly different compared to control, ^#^*P *< 0.05, ^### ^*P *< 0.0001 significantly different compared to the fenofibrate group.

Plasma levels of leptin were significantly lower in both fenofibrate (*P *= 0.02) and pioglitazone groups (*P *= 0.03) compared to controls (Figure 1A). In femoral bone, the leptin concentration was lower in pioglitazone rats (*P *= 0.003 vs. control and *P *= 0.03 vs. fenofibrate group), while the fenofibrate group did not differ from controls (Figure 1B). Plasma levels of adiponectin were significantly lower in the fenofibrate group compared to controls (*P *< 0.0001) (Figure 1C), while the adiponectin levels were significantly higher in the pioglitazone group both compared to controls (*P *< 0.0001), and to the fenofibrate group (*P *< 0.0001) (Figure 1C). There were no differences in the adiponectin levels in femoral bone between the groups (Figure 1D).

### Insulin, osteocalcin, OPG and bone resorption marker measurements

There were no differences in plasma levels of insulin between the groups (controls: 1.42 ± 0.66 ng/ml, fenofibrate group: 1.35 ± 0.69 ng/ml and pioglitazone group: 1.32 ± 0.52 ng/ml). Plasma levels of osteocalcin were significantly higher in the fenofibrate group compared to controls (*P *= 0.012), while the pioglitazone group did not differ from controls (Figure 1E). Plasma OPG levels were significantly lower in the pioglitazone group compared to controls (*P *= 0.009), while the fenofibrate group did not differ from controls (Figure 1F). There were no differences in plasma levels of bone resorption marker (fragments of collagen type I) between the groups (data not shown).

### Fenofibrate stimulates mRNA expression of osteoblast differentiation markers

After 12 days of stimulation, fenofibrate (0.1 μM) significantly increased the mRNA expression of ALP, BSP, CD44, collagen 1, and osteocalcin in MC3T3-E1 cells (Table 5). Pioglitazone (0.1 μM) increased the relative CD44 mRNA expression after 12 days of stimulation, but none of the other osteoblast differentiation genes examined (Table 5). Other concentrations of both fenofibrate and pioglitazone (1.0 μM and 10 μM) were examined, with similar patterns. The early adipocyte differentiation marker lipoprotein lipase (LPL) mRNA was not expressed at day 0, but was low and equally expressed in all cells at day 4, 8 and 12 of stimulation (Table 5). PPARα mRNA expression was significantly enhanced compared to control by both fenofibrate and pioglitazone after 4 days, and also after 8 and 12 days by fenofibrate. There was, however, no detectable expression of PPARα mRNA in pioglitazone stimulated cells after 12 days. PPARγ expression was equally expressed in all cells throughout the stimulation period (Table 5).

**Table 5:** Relative mRNA expression of selected genes in MC3T3-E1 cells

DAY		Alcalic phosphatase (ALP)	Bone Sialoprotein	CD44	Collagen 1	Lipoprotein lipase (LPL)
0		1.00 ± 0.05	1.00 ± 0.44	1.00 ± 0.30	1.00 ± 0.10	No detectable expression

4	Control	1.00 ± 0.15	1.00 ± 0.18	1.00 ± 0.03	1.00 ± 0.24	1.00 ± 0.51
	Fenofibrate 0.1 μM	0.62 ± 0.11	0.88 ± 0.23	1.05 ± 0.17	0.92 ± 0.18	1.12 ± 0.23
	Pioglitazone 0.1 μM	1.06 ± 0.19	1.40 ± 0.62	1.52 ± 0.06	1.17 ± 0.19	0.90 ± 0.01

8	Control	1.00 ± 0.27	1.00 ± 0.54	1.00 ± 0.17	1.00 ± 0.03	1.00 ± 0.12
	Fenofibrate 0.1 μM	1.47 ± 0.04	2.19 ± 0.24	1.09 ± 0.06	0.76 ± 0.0	0.81 ± 0.01
	Pioglitazone 0.1 μM	1.40 ± 0.16	2.07 ± 1.45	0.81 ± 0.04	0.34 ± 0.04	1.00 ± 0.06

12	Control	1.00 ± 0.12	1.00 ± 0.50	1.00 ± 0.03	1.00 ± 0.05	1.00 ± 0.58
	Fenofibrate 0.1 μM	3.91 ± 0.20**	7.40 ± 0.82*	5.04 ± 0.03***	6.52 ± 0.78**	0.97 ± 0.14
	Pioglitazone 0.1 μM	2.40 ± 0.75	3.94 ± 1.29	2.42 ± 0.11**	2.38 ± 1.00	1.93 ± 1.36

DAY		Osteocalcin	Osteopontin	PPARα	PPARγ	

0		1.00 ± 0.23	1.00 ± 0.14	1.00 ± 0.51	1.00 ± 0.04	

4	Control	1.00 ± 0.34	1.00 ± 0.02	1.00 ± 0.05	1.00 ± 0.30	
	Fenofibrate 0.1 μM	0.54 ± 0.19	0.99 ± 0.14	2.51 ± 0.43*	0.86 ± 0.24	
	Pioglitazone 0.1 μM	1.38 ± 0.05	0.76 ± 0.02	1.76 ± 0.02*	1.06 ± 0.09	

8	Control	1.00 ± 0.20	1.00 ± 0.30	1.00 ± 0.21	1.00 ± 0.06	
	Fenofibrate 0.1 μM	0.54 ± 0.11	0.73 ± 0.04	2.83 ± 0.40*	0.93 ± 0.05	
	Pioglitazone 0.1 μM	0.67 ± 0.02	0.34 ± 0.02	0.98 ± 0.08	2.23 ± 0.36*	

12	Control	1.00 ± 0.10	1.00 ± 0.71	1.00 ± 0.04	1.00 ± 0.31	
	Fenofibrate 0.1 μM	2.34 ± 0.01**	2.38 ± 0.39	1.43 ± 0.23	1.20 ± 0.06	
	Pioglitazone 0.1 μM	1.89 ± 0.27	1.82 ± 2.32	No detectable expression	1.98 ± 0.61	

PPAR = peroxisome proliferator activated receptor

Data is in presented as mean ± SD, calculated from standard curves and related to housekeeping gene and normalized to controls. Data is from three biological replicates, *P < 0.05, **P < 0.01, ***P < 0.001 when compared to controls at same time (two-tailed student t-test).

### Fenofibrate stimulates proliferation of and OPG release from preosteoblasts

**Figure 2:**
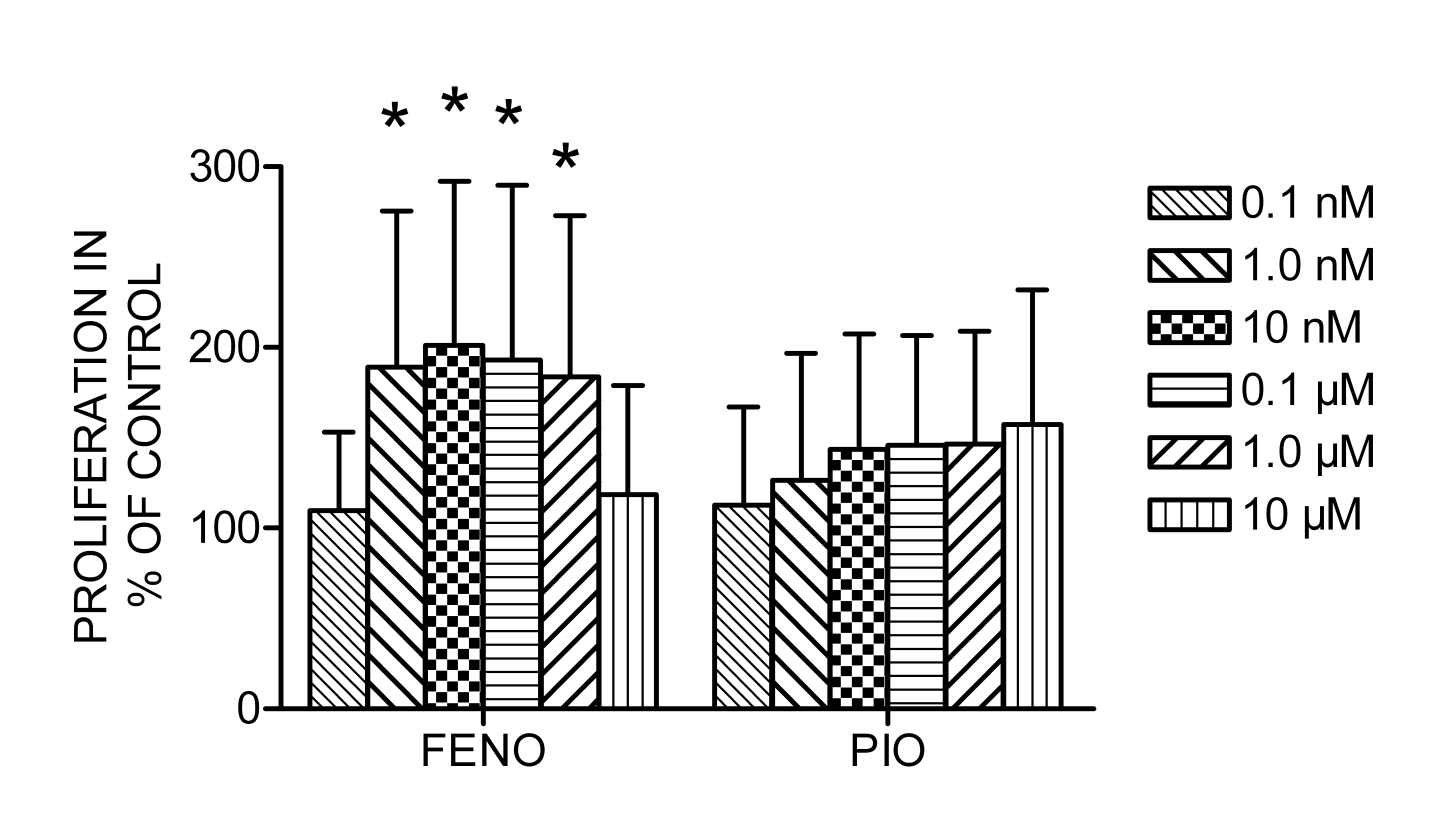
**Effect of the PPARα agonist fenofibrate and the PPARγ agonist pioglitazone on proliferation in MC3T3-E1 preosteoblast cells**. Data is presented in mean ± SD in % of control (unstimulated cells) from four parallels in each experiment, and the figure represents data from four different experiments. *P < 0.05 significantly different compared to control.

Fenofibrate (1.0 nM – 1.0 μM) increased proliferation of MC3T3-E1 preosteoblasts, whereas pioglitazone had no effect (Figure 2).

**Figure 3:**
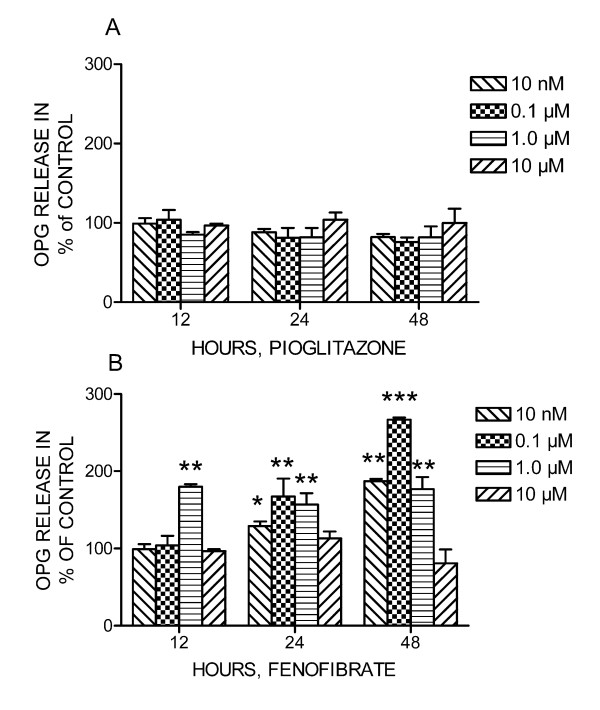
**Effect of PPARα agonist fenofibrate (A) and PPARγ agonist pioglitazone (B) on osteoprotegerin (OPG) release from MC3T3-E1 preosteoblast cells**. Amount of OPG is related to the amount of total protein in each sample. Data is presented in mean ± SD in % of control (unstimulated cells) from two parallels, and the figure represents data from four different experiments. *P < 0.05, **P < 0.01, ***P < 0.001 significantly different compared to control.

MC3T3-E1 cells were treated with fenofibrate and pioglitazone (1.0 nM to 10 uM) for 12, 24, 48 and 72 h to study the effect on release of OPG and RANKL. Fenofibrate increased OPG release in a dose-dependent manner after 12–48 h stimulation (*P *< 0.05) (Figure 3A). In contrast, pioglitazone had no effect on OPG release from MC3T3-E1 cells (Figure 3B). Neither fenofibrate nor pioglitazone induced significant changes in RANKL secretion from MC3T3-E1 cells compared to untreated cells (data not shown).

### Osteoclast differentiation and activity

We found no effect of fenofibrate or pioglitazone on either differentiation or activity of primary human osteoclasts.

## Discussion

TZD use has been shown to increase bone loss and fracture risk in women with diabetes mellitus type 2 [17], decrease BMD in diabetic men [33], and also to decrease bone formation and BMD in healthy postmenopausal women [19]. In the present study we have studied the skeletal effects of pioglitazone which is a selective PPARγ agonist [8] and fenofibrate which belongs to the class of fibrates and is mainly a PPARα agonist [34]. Both substances are well described and are used in the clinic for treatment of type 2 diabetes mellitus and hypertriglyceridemia, respectively [8,9]. We report effects differentiating the PPARα agonist fenofibrate from the PPARγ agonist pioglitazone in intact female rats. Our data represent to our knowledge the first evidence of positive skeletal effects of fenofibrate. Femoral BMD measured by DXA was significantly increased in female rats receiving fenofibrate. We have previously shown that Wyeth 14643, which is a model substance for PPARα agonists, leads to a similar increment in BMD [35], supporting that this effect is mediated through PPARα activation. The increase in BMD following administration of fenofibrate was associated with a decrease in medullary area. Changes in marrow cavity area could be the result of combined effects on bone resorption and formation at the endosteal surface. Our results do, however, not permit differentiation between these effects. These changes were not reflected in other significant histomorphometric differences, nor improved mechanical properties of bone.

Rats treated with pioglitazone exhibited the opposite skeletal response. They had a significantly lower whole body BMD and BMC, and tended to have a lower femoral BMD. These data were further corroborated by histomorphometry showing decreased trabecular bone volume and increased medullary area in femur, suggesting increased endosteal resorption. Moreover, these changes were reflected in a decline in ultimate bending moment and energy absorption in the femoral shaft indicating increased skeletal fragility. The findings of this study support previous reports demonstrating a negative impact on the skeleton of rodents treated with the PPARγ agonist rosiglitazone [14-16]. Taken together, these data indicate positive skeletal effects of PPARα agonists, while PPARγ activation has a net negative effect on the skeleton. However, PPAR-independent effects can not be ruled out.

In addition to the effects on BMD, we observed changes in fat mass and lean mass. In accordance with previous studies [22,23], an increase in fat mass was found in animals given pioglitazone. No difference in body weight was found between the groups.

Leptin has emerged as a significant factor in the regulation of bone mass, as reviewed by Reid *et al*. [36]. We observed a decrease in plasma leptin levels in both fenofibrate and pioglitazone rats, which is in accordance with previous studies. Adipose tissue is the strongest determinant of serum leptin levels. In a study by Damci *et al*., fenofibrate treatment of type 2 diabetics with hypertriglyceridemia was associated with lower serum leptin levels in spite of unchanged body mass index during the study [37]. Several studies describe a suppression of serum leptin in connection with TZDs use in spite of the increased fat mass [22,23,38]. In our study leptin levels in the protein fraction of crushed femurs were unchanged in fenofibrate rats compared to controls, while significantly reduced in animals receiving pioglitazone.

In accordance with previous studies on TZDs, adiponectin levels were elevated in the pioglitazone group [24,25], while a significant decrease was observed in the fenofibrate rats. This is in contrast to studies in humans where administration of fibrates is associated with a rise in plasma adiponectin [26]. Adiponectin-adenovirus treatment increased trabecular bone mass in mice [39], whereas Reid *et a*l. report increased bone mass in adiponectin knock-out mice [40]. Increasing levels of adiponectin are found to be associated with a decrease in BMD in postmenopausal women [41]. Data on the effects of the adipokines leptin and adiponectin on bone are conflicting, and it is difficult to interpret the significance of the observed changes in our study.

The changes in fat mass were associated with reciprocal effects on lean mass, with the pioglitazone group showing significantly lower lean mass, and the fenofibrate rats significantly higher lean mass. The increase in liver weight in the fenofibrate group is a well known effect of PPARα activation [42], and contributes to the elevated lean mass in this group. After correction for this, lean mass is, however, still higher in the fenofibrate group than in the two others. Muscle plays a critical role in bone growth and remodeling because the greatest loads on bone arise from muscle contractions. Therefore, the differences in BMD between the groups could partly be explained by various effects on lean mass. This is supported by a positive correlation between lean mass and BMD, as well as cortical area.

Enhanced osteoclastogenesis leading to bone loss is the main etiological factor in osteoporosis development. Osteoclast differentiation is regulated by the coordinated synthesis and actions of the cytokines RANKL and OPG produced by bone marrow stromal cells and osteoblasts [43,44]. In the present study fenofibrate, but not pioglitazone, stimulated OPG release from MC3T3-E1 cells while RANKL release was unaffected by both agents. Since OPG is an important inhibitor of osteoclast differentiation via binding of RANKL, these findings indicate an antiresorptive effect of fenofibrate. Our findings are supported by a newly published study showing that fenofibrate increases plasma OPG in humans [45]. The observed decrease in cross-sectional medullary area may be explained by this effect. It has previously been shown that fenofibrate also inhibits osteoclast differentiation directly [11]. We were, however, not able to confirm those findings in human preosteoclasts.

Plasma OPG levels were significantly lower in rats treated with pioglitazone. Pioglitazone did, however, not affect the release of OPG or RANKL from MC3T3-E1 cells, and no effect on osteoclast differentiation or activity was observed. Enhanced marrow adipogenesis and bone resorption have also been described in estrogen-deprived rats treated with rosiglitazone [16]. The increased medullary area observed in our study also suggests an increased endosteal resorption.

Previous studies have concluded that rosiglitazone affects the skeleton primarily via a negative impact on bone formation. Rosiglitazone activates adipocyte differentiation and inhibits osteoblast differentiation [28], and an increase in fat content and number of adipocytes has been shown in trabecular bone after administration of rosiglitazone [14]. These effects seem to be mediated via activation of PPARγ2. Rzonca *et al*. found that expression of the osteoblast specific marker genes runx2 and alpha1(I) collagen in the tibia was decreased in mice receiving rosiglitazone [14], and Kha *et al*., have demonstrated that the PPARγ agonist troglitazone induces adipogenesis in expense of osteogenesis in mesenchymal stem cells [46]. Based on these data one would expect pioglitazone to interfere with osteoblast formation in a similar manner. In the present study, however, we were unable to show any effects of pioglitazone on osteoblast differentiation in the mouse preosteoblast cell line MC3T3-E1. This might be explained by the fact that these cells are already committed to the osteoblastic pathway

*In vivo *studies have demonstrated that heterozygous PPARγ-deficient mice have enhanced bone mass as a result of increased osteoblastogenesis [47], and that the PPARγ^hyp/hyp ^mouse model, which does not express PPARγ (1 nor 2), has increased bone mass due to favoured osteogenic, rather than adipogenic, differentiation of mesenchymal precursor cells [48] Our *in vivo *data suggest an effect of the PPARγ agonist mainly on bone resorption. We have, however, only structural histomorphometric data, and are not able to differentiate between increased bone resorption and decreased bone formation.

In contrast, we found that fenofibrate stimulated osteoblast differentiation, as well as proliferation. The pronounced stimulation of type I collagen mRNA, denotes increased bone matrix formation. This effect needs to be confirmed by protein measurements. Our data correspond with previous reports, demonstrating that the PPARα agonist Wyeth 14643 induces osteoblast maturation of preosteoblasts, indicating a positive effect of PPARα agonists on bone formation [4]. This notion is further supported by the increased levels of plasma osteocalcin in fenofibrate rats in our study. Theoretically, the decrease in marrow cavity area could be a result of combined effects on bone resorption and formation at the endosteal surface.

## Conclusion

We show opposite skeletal effects of PPARα and γ agonists in intact female rats. We report for the first time that fenofibrate increases femoral BMD and decreases medullary area in femur. This appears to be associated with changes in cortical bone, since trabecular bone was unchanged. The stimulatory effect of fenofibrate on osteoblast proliferation and differentiation, as well as OPG release, constitutes also new findings. The observed negative skeletal effects of pioglitazone confirm previous findings with rosiglitazone in rodents. These findings are of particular interest in light of recent human studies showing enhanced bone loss and increased fracture risk in TZDs users [17-19,33,49]. It is conceivable that further studies on the effects of PPAR agonists may give new insights in the regulation of bone mass and pathogenesis of osteoporosis, insights, which may potentially offer new treatment options.

## Competing interests

Eli Lilly, Norway supplied pioglitazone used in this study. The company has, however, not been involved in the planning or execution of the study, and is not involved in the interpretation, writing or publication of the final results. The work is also funded by Nycomed Pharma AS, Norway, which is a commercial company with no interests in PPAR agonists. The trial has been run independent of these companies.

## Authors' contributions

US planned and coordinated the study, and drafted the manuscript, AKS carried out cell studies, molecular genetic studies, immunoassays, performed statistical analyses and contributed in writing. BIG and LN did the biomechanical investigations, KJO was responsible for the histomorphometry and Rolf Berge carried out immunoassays. LT performed cell studies and molecular genetic studies and JER participated i immunoassays, statistical analyses and writing. All authors read and approved the final manuscript.

## Pre-publication history

The pre-publication history for this paper can be accessed here:


